# Large language models for depression prediction

**DOI:** 10.1073/pnas.2409757121

**Published:** 2024-07-25

**Authors:** Yu Wang

**Affiliations:** ^a^Fudan Institute for Advanced Study in Social Sciences, Fudan University, Shanghai 200433, China

In a recent study on the moderating effects of race on the relationship between natural language and depression, Rai et al. (1) find that machine learning models trained using the language of Black individuals performed worse than models trained using the language of White individuals (Table 1). Given these findings, the authors stress the importance of better understanding how race influences the expression of depression, “before language-based models for detecting psychological phenomena are integrated into clinical practice.” In this letter, we raise two questions regarding the study by Rai et al. (1).

**Table 1.** Reproduced from Rai et al. (1): Models trained on White individuals’ language showed strong correlation with White test set, whereas the models have a weak correlation with Black test set

	White test set (1)	Black test set (2)
Feature set: 1 to 3 gm, LIWC categories and Latent Dirichlet Allocation topics		
(1) M_white_	0.392 (0.000)	0.132 (0.006)
(2) M_black_	0.204 (0.000)	0.126 (0.009)
Feature set: BERT embeddings (Layer 11 and 12)		
(3) M_white_	0.347 (0.000)	0.104 (0.031)
(4) M_black_	0.161 (0.001)	0.058 (0.225)

First, observing that models trained on the language of Black individuals performed poorly as compared with models trained on that of White individuals, Rai et al. (1) suggest that either this is because “depression may not manifest in language for Black individuals” or that “different language markers not examined here, such as other word categories or paralinguistic features (e.g., tone, speech rate), could relate to depression among Black individuals.” These hypotheses are supported when we compare Row 1, Column 1 with Row 2, Column 2 or compare Row 3, Column 1 with Row 4, Column 2. Nonetheless, these hypotheses fail to explain why models trained on the language of Black individuals perform better in the White test set than in the Black test set (Rows 2 and 4). We normally expect models to perform better on in-domain data than on out-of-domain data (2). Note that this is orthogonal to the hypothesis of the language of Black individuals containing different language markers or no markers at all. Unfortunately, key information on the experiment is missing, including the validation metrics, size of the training set, size of the test set, and samples of the raw datasets. Without this crucial information, it is difficult to pin down the exact cause behind the results in Row 2, Column 1 and in Row 4, Column 1. This inevitably casts doubt over the validity of the authors’ results.

Second, in addition to training regression models with such language features as Linguistic Inquiry of Word Count (LIWC) (3), Rai et al. (1) train a separate set of models using BERT embeddings as input (4). Upon noticing that models trained on the embeddings of Black individuals do not correlate significantly with Black test set, the authors suggest that this could be because “depression may not manifest in language for Black individuals.” We contend that the suggestion is premature. Running regressions using embeddings from a BERT-base model is suboptimal in terms of model performance (5). A substantially more effective approach would be fine-tuning the pretrained BERT model rather than using it as a frozen embedding model (6, 7). Furthermore, the authors could utilize a larger and more performant version of the BERT model, or opt for RoBERTa, instead of the BERT-base model. Until such models are adequately trained and tested, which could well outperform both embedding-based and language marker-based models, it is not reasonable to conclude that “depression may not manifest in language for Black individuals.”
